# Mechanical Unloading Inhibits Osteoblast Differentiation via Downregulation of OGT-Mediated O-GlcNAcylation

**DOI:** 10.3390/cimb48090961

**Published:** 2026-09-20

**Authors:** Xiran Zhao, Junfei Zhang, Zhikui Li, Quan Sun, Liqun Xu, Tong Xue, Jiangdong Zhao, Xian Guo, Ru Zhang, Xuan Xie, Zhijun Yan, Zebing Hu, Shu Zhang, Fei Shi

**Affiliations:** 1 The Key Laboratory of Aerospace Medicine, Ministry of Education, The Fourth Military Medical University, Xi’an 710032, China; 2 Department of Gastroenterology and Endocrinology, Western Theater Air Force Hospital of PLA, Chengdu 610065, China

**Keywords:** O-GlcNAcylation, O-GlcNAc transferase, mechanical unloading, osteoblast differentiation

## Abstract

Prolonged spaceflight and sustained bed rest induce mechanical unloading, leading to disuse osteoporosis and an elevated risk of fractures. Although post-translational modifications are increasingly recognized as key contributors to the pathogenesis of disuse bone loss, the functional role and underlying molecular mechanisms of O-linked N-acetylglucosaminylation (O-GlcNAcylation) remain poorly understood. Here, we demonstrate that mechanical unloading via 2D clinorotation downregulates the levels of O-GlcNAc transferase (OGT) and global protein O-GlcNAcylation in MC3T3-E1 cells, whereas osteogenic induction elicits the opposite effect. Both small interfering RNA (siRNA) targeting OGT and pharmacological inhibition using OSMI-1 recapitulated unloading-induced deficits, significantly impairing osteogenic differentiation and matrix mineralization. Conversely, OGT overexpression or inhibition of O-GlcNAcase (OGA) with Thiamet-G enhanced these processes. Importantly, OGT re-expression partially reversed the deficits caused by mechanical unloading. Notably, exogenous elevation of global O-GlcNAcylation levels via Thiamet-G treatment even after OGT knockdown also partially restored osteogenic capacity. Together, these findings establish the OGT/O-GlcNAcylation axis as a critical regulator of unloading-induced suppression of osteogenesis and identify it as a promising therapeutic target for disuse osteoporosis.

## 1. Introduction

Unlike primary osteoporosis caused by aging and gonadal dysfunction, disuse osteoporosis arises from prolonged mechanical unloading of the skeleton, which subsequently induces systemic bone loss and microarchitectural deterioration [1,2]. This form of secondary osteoporosis commonly occurs during long-term spaceflight, spinal cord injury, or prolonged bed rest, and is also associated with complications such as fractures, soft tissue calcification, urolithiasis, and vascular atherosclerosis [2,3]. Notably, bone loss induced by microgravity in space occurs at a faster rate and is more difficult to recover from than that observed in terrestrial settings. Current prevention and treatment strategies primarily fall into two categories: pharmacological interventions and physical therapies [4,5]. Commonly prescribed anti-osteoporotic drugs fall into two categories: antiresorptive agents (including bisphosphonates and denosumab) and anabolic agents (including teriparatide, abaloparatide, and romosozumab). Long-term bisphosphonate therapy is associated with rare but serious adverse events, notably atypical femoral fractures and osteonecrosis of the jaw. Discontinuation of denosumab carries a well-documented risk of rapid bone loss and incident vertebral fractures. Among anabolic agents, teriparatide and abaloparatide are associated with higher discontinuation rates due to treatment-emergent adverse effects, whereas romosozumab has raised cardiovascular safety concerns in patients with established ischemic heart disease. Collectively, existing anti-osteoporotic drugs face major clinical limitations, as they often cause severe side effects, show high inter-individual variability in efficacy, and lead to poor patient compliance during long-term treatment [6,7,8,9]. Although physical therapies, including exercise, vibration, and electrical stimulation, are non-invasive and demonstrate established physiological benefits, their clinical effectiveness remains inconsistent across populations, long-term patient adherence is suboptimal, and standardized treatment protocols are absent [4,5]. Furthermore, they frequently fail to prevent or reverse disuse-induced bone loss as monotherapies, especially in patients with severe mobility impairments. The pathogenesis of disuse bone loss involves multifaceted interactions, and the underlying mechanisms are far more complex than a simple perturbation of a single signaling pathway. In particular, the molecular regulatory networks governing the suppression of bone formation under mechanical unloading conditions remain largely unelucidated. Therefore, there is an urgent need to dissect the biological essence of gravitational unloading at the molecular level and to systematically identify the key signaling pathways and critical regulatory nodes involved. Such investigations will not only facilitate the discovery of novel mechanisms governing bone metabolism, but also lay the foundation for developing innovative therapeutic strategies against bone loss to support future space missions.

Bone metabolism relies on a tight balance between osteoblast-mediated bone formation and osteoclast-mediated bone resorption, which is essential for maintaining bone mass and structural integrity [2,10,11]. Osteoblasts play a pivotal role in the process of bone formation. Accumulating evidence indicates that mechanical unloading suppresses osteoblast differentiation and consequently impairs bone formation [12,13,14,15]. Previous studies have investigated the underlying mechanisms from multiple perspectives, including mechanosensors [16,17], signal transduction pathways [18,19], epigenetic modifications [13,20], and post-translational modifications [21,22,23,24,25,26,27]. Despite these insights, the exact molecular mechanisms through which mechanical unloading inhibits osteoblast differentiation remain incompletely understood.

O-linked N-acetylglucosaminylation (O-GlcNAcylation) is a reversible post-translational modification involved in many biological processes, such as gene expression, protein stability, and signal transduction [28,29,30,31]. This modification is controlled by two enzymes: O-GlcNAc transferase (OGT) adds GlcNAc to target proteins, while O-GlcNAcase (OGA) removes it [28]. In recent years, the regulatory roles of O-GlcNAcylation in bone remodeling have attracted growing interest [32,33,34]. Prior studies have documented marked elevations in the O-GlcNAc modification levels of numerous proteins during osteoblast differentiation [27,33]. Zhang et al. [33] reported that the O-GlcNAcylation of FOXO3 is essential for directing the osteogenic differentiation of bone marrow mesenchymal stem cells. With regard to osteoporosis, Gu et al. [35] demonstrated that high glucose in diabetic patients leads to abnormal O-GlcNAcylation, which reduces BMP2-induced osteogenic differentiation in C2C12 cells. Conversely, Du et al. [36] showed that OGT expression and global O-GlcNAcylation levels were reduced in ovariectomized mice, whereas pharmacological inhibition of OGA to boost O-GlcNAc levels promoted osteoblast differentiation and alleviated bone loss induced by ovariectomy (OVX). Zhang et al. [37] further revealed that O-GlcNAcylation of LEP facilitates the inactivation of the NF-κB pathway through reduction of LEP protein abundance, thereby suppressing cellular senescence and promoting osteogenic differentiation in C3H10T1/2 cells. Additionally, mechanical compression has been reported to induce chondrocyte hypertrophy in temporomandibular joint degeneration by regulating Runx2 O-GlcNAcylation [38]. Despite these findings that implicate OGT and O-GlcNAcylation in various osteoporotic conditions and mechanical stimuli, little is known about how OGT and O-GlcNAcylation specifically affect disuse osteoporosis and osteoblast differentiation, or what downstream molecular events are involved. Addressing these outstanding questions will not only deepen our knowledge of OGT and O-GlcNAcylation in the regulation of bone metabolism, but may also uncover novel therapeutic targets for the prevention and management of disuse bone loss.

In this study, we investigated the role of OGT and O-GlcNAcylation in osteoblast differentiation under mechanical unloading. By employing a 2D clinostat model and genetic and pharmacological manipulations, we aimed to determine whether OGT-mediated O-GlcNAcylation regulates osteoblast differentiation and mineralization, and to explore its potential as a therapeutic target for disuse osteoporosis. This study may provide mechanistic insights into how mechanical unloading suppresses bone formation and may contribute to the development of novel strategies for the prevention and treatment of disuse osteoporosis.

## 2. Materials and Methods

### 2.1. Cell Culture and In Vitro Differentiation

The murine pre-osteoblast cell line MC3T3-E1 was purchased from the Cell Bank of the Chinese Academy of Sciences (Shanghai, China). Cells were maintained in α-MEM basal medium (Gibco, Grand Island, NY, USA) supplemented with 10% fetal bovine serum (FBS; Gibco, Grand Island, NY, USA) and 1% penicillin–streptomycin (Gibco, Grand Island, NY, USA), under standard conditions of 37 °C, 5% CO_2_, and 95% relative humidity. Osteogenic differentiation was induced by culturing MC3T3-E1 cells in osteogenic induction medium—α-MEM complete medium supplemented with 100 nM dexamethasone, 50 μg/mL ascorbic acid, and 10 mM β-glycerophosphate—for up to 21 days. Experiments were conducted using cells between passages 4 and 8, and all assays were performed in triplicate.

### 2.2. Two-Dimensional Clinorotation

The two-dimensional clinostat developed by the China Astronaut Research and Training Center was employed to establish a mechanical unloading model. MC3T3-E1 cells were seeded at a density of 5 × 10^5^ cells per 25-cm^2^ rotation-specific culture flask. To minimize fluid shear stress, each flask was completely filled with culture medium and sealed, with all air bubbles carefully removed. Cells were then mounted on the clinostat and rotated around a horizontal axis at a constant speed of 24 r/min, which was selected to minimize hydrodynamic perturbations and significantly reduce convective motion and associated shear stresses. The control group was maintained under identical environmental conditions but without rotation. Both rotating and control groups underwent identical handling, ensuring observed changes reflect mechanical unloading alone.

### 2.3. RNA Extraction and Quantitative Real-Time Polymerase Chain Reaction (qRT-PCR)

Total RNA was isolated from harvested cells using RNAiso Plus reagent (TaKaRa, Tokyo, Japan). Prime Script™ RT Master Mix reagent kit (TaKaRa, Tokyo, Japan) was used to convert the mRNA into cDNA according to the manufacturer’s instructions. Real-time quantitative PCR (qRT-PCR) was performed on a CFX96 detection system (Bio-Rad, Hercules, CA, USA) using SYBR Premix Ex Taq ^TM^ II (TaKaRa, Tokyo, Japan). The relative expression levels of target genes were normalized to the reference gene GAPDH, and fold changes were calculated using the 2^−ΔΔCt^ method. All primer sequences are listed in Table 1.

### 2.4. Protein Isolation and Western Blotting Assay

For protein extraction, MC3T3-E1 cells were harvested using a cell scraper and lysed in M-PER mammalian protein extraction reagent (Thermo Fisher Scientific, Waltham, MA, USA) supplemented with protease and phosphatase inhibitors (NCM Biotech, Suzhou, China). The lysates were briefly sonicated and centrifuged at 12,000× *g* for 10 min at 4 °C to remove insoluble debris. The supernatant was collected, and protein concentrations were determined using the Pierce™ BCA assay kit (Thermo Fisher Scientific, Waltham, MA, USA) with bovine serum albumin as the standard.

Equal amounts of protein (30 µg per lane) were mixed with loading buffer, separated by 10% SDS-PAGE, and electrotransferred onto PVDF membranes (Millipore, Billerica, MA, USA) in an ice-cooled transfer apparatus. Following transfer, the membranes were blocked with 5% skimmed milk in Tris-buffered saline containing 0.1% Tween-20 (TBST) for 2 h at room temperature, and then probed overnight at 4 °C with primary antibodies against the proteins of interest. The following primary antibodies were used: GAPDH (1:50,000, 60004-1-Ig; Proteintech, Chicago, IL, USA), OGA (1:5000, 14711-1-AP; Proteintech, Chicago, IL, USA), OGT (1:1000, #24083S; Cell Signaling Technology, Danvers, MA, USA), Col1al (1:1000, A1352; Abclonal, Wuhan, China), Runx2 (1:1000, #12556S; Cell Signaling Technology, Danvers, MA, USA), Ocn (1:1000, ab93876, Abcam, Cambridge, UK), O-GlcNAc (RL2, 1:1000, MA1-072, Thermo Fisher Scientific, Waltham, MA, USA). After extensive washing with TBST, the membranes were incubated with either an HRP-conjugated goat anti-mouse antibody (1:5000, ZB-2305; ZSGB-BIO, Beijing, China) or an anti-rabbit secondary antibody (1:5000, ZB-2301; ZSGB-BIO, Beijing, China) for 1.5 h at room temperature. Immunoreactive bands were visualized using an enhanced chemiluminescence substrate (Thermo Fisher Scientific, Waltham, MA, USA) and captured with a Tanon-4200 (Tanon Science and Technology Ltd, Shanghai, China) imaging system. Band intensities were quantified using ImageJ 1.52a software, with GAPDH serving as the loading control for normalization.

### 2.5. Cell Transfection

Cells were seeded in 6-well plates and transfected at 30–50% confluence using Lipofectamine 2000 (Thermo Fisher Scientific, Waltham, MA, USA) according to the manufacturer’s protocol. For gene silencing, cells were treated with 80 nM si-OGT-1, si-OGT-2, or the corresponding negative control (GenePharma, Shanghai, China). For overexpression experiments, cells were transfected with 40 nM pcDNA3.1-OGT plasmids or empty pcDNA3.1 vector plasmids (GenePharma, Shanghai, China) using Lipofectamine RNAiMAX (Thermo Fisher Scientific, Waltham, MA, USA). Following transfection, cells were maintained for 48 h before subsequent analyses.

For osteogenic differentiation assays, the transfection schedule was adapted according to the experimental endpoint. For ALP staining, a single transfection was performed prior to osteogenic induction, and ALP staining was conducted on day 7 of differentiation. For Alizarin Red S staining, cells were transfected prior to osteogenic induction and re-transfected on day 7 to maintain OGT knockdown or overexpression throughout the mineralization phase, followed by staining on day 21. Identical transfection reagents and conditions were used for both rounds. The sequences of the siRNAs and negative controls are provided in Table 2.

### 2.6. Alkaline Phosphatase Staining (ALP Staining)

For ALP staining, the BCIP/NBT Alkaline Phosphatase Color Development Kit (Beyotime, Shanghai, China) was employed. After removing the culture medium, cells were washed twice with PBS and then fixed with 4% paraformaldehyde at room temperature for 30 min, followed by three rinses with PBS (5 min each). The BCIP/NBT working solution was added to each well and incubated for 30 min at room temperature under light-protected conditions. After removal of the working solution, the reaction was terminated by two washes with distilled water. Digital images were then captured.

### 2.7. Alizarin Red S Staining

To evaluate matrix mineralization, MC3T3-E1 cells were cultured in osteogenic induction medium for 21 days. After removing the culture supernatant, the cell layers were gently rinsed with DPBS and then fixed with pre-chilled 70% ethanol for 1 h at 4 °C. Following three washes with distilled water, the fixed cells were stained with 40 mM Alizarin Red S solution (ARS; Sigma, St. Louis, MO, USA) for 15 min at room temperature to visualize calcium deposits. To minimize nonspecific background staining, the stained monolayers were treated with DPBS for 15 min and subsequently washed thoroughly with distilled water. Mineralized nodules were observed under a microscope with a 10× objective lens and imaged using a digital camera under bright-field illumination at room temperature. Identical imaging conditions were maintained across all experimental groups.

### 2.8. Rescue Experiments

MC3T3-E1 cells were cultured in a rotation-specific culture flask, and transfection was initiated upon reaching approximately 60% confluence. After 6–8 h, the medium was refreshed, and the flasks were sealed following removal of air bubbles. Subsequently, cells were mounted on the two-dimensional clinostat for 48 h. Four experimental groups were designated: Con, Clino, Clino + pcDNA3.1, and Clino + pcDNA3.1-Ogt.

### 2.9. CCK-8 Assay

Cell viability was determined using the Cell Counting Kit-8 (CCK-8; C6005, NCM Biotech, Suzhou, China) according to the manufacturer’s instructions. MC3T3-E1 cells were seeded into 96-well plates at a density of 5 × 10^3^ cells per well and maintained overnight for attachment. After OSMI-1 or Thiamet-G treatment, 10 μL of CCK-8 solution was added to each well, and the plates were incubated at 37 °C for 1 h. The absorbance was measured at 450 nm using a microplate reader.

### 2.10. OSMI-1 and Thiamet-G Treatment

Cells were treated with 10 μM OSMI-1 (HY-119738, MCE, South Brunswick, NJ, USA), 25 μM Thiamet-G (HY-12588, MCE, South Brunswick, NJ, USA), or an equivalent volume of DMSO as a vehicle control for the indicated durations. For the Thiamet-G rescue experiment, cells were first transfected and then, 24 h later, exposed to Thiamet-G for an additional 24 h to modulate global O-GlcNAcylation levels.

### 2.11. Statistical Analysis

All statistical evaluations were carried out with GraphPad Prism 10.1.2 (GraphPad Software, La Jolla, CA, USA). Intergroup comparisons between two groups were made using the Student’s *t*-test, and one-way ANOVA followed by Holm–Šídák correction was applied for multiple group comparisons. Data are shown as the mean ± standard deviation from at least three independent biological experiments, each performed with three technical replicates. Statistical significance was defined as *p* < 0.05.

## 3. Results

### 3.1. OGT and Global O-GlcNAcylation Levels Are Positively Correlated with Osteogenic Differentiation

**Figure 1:**
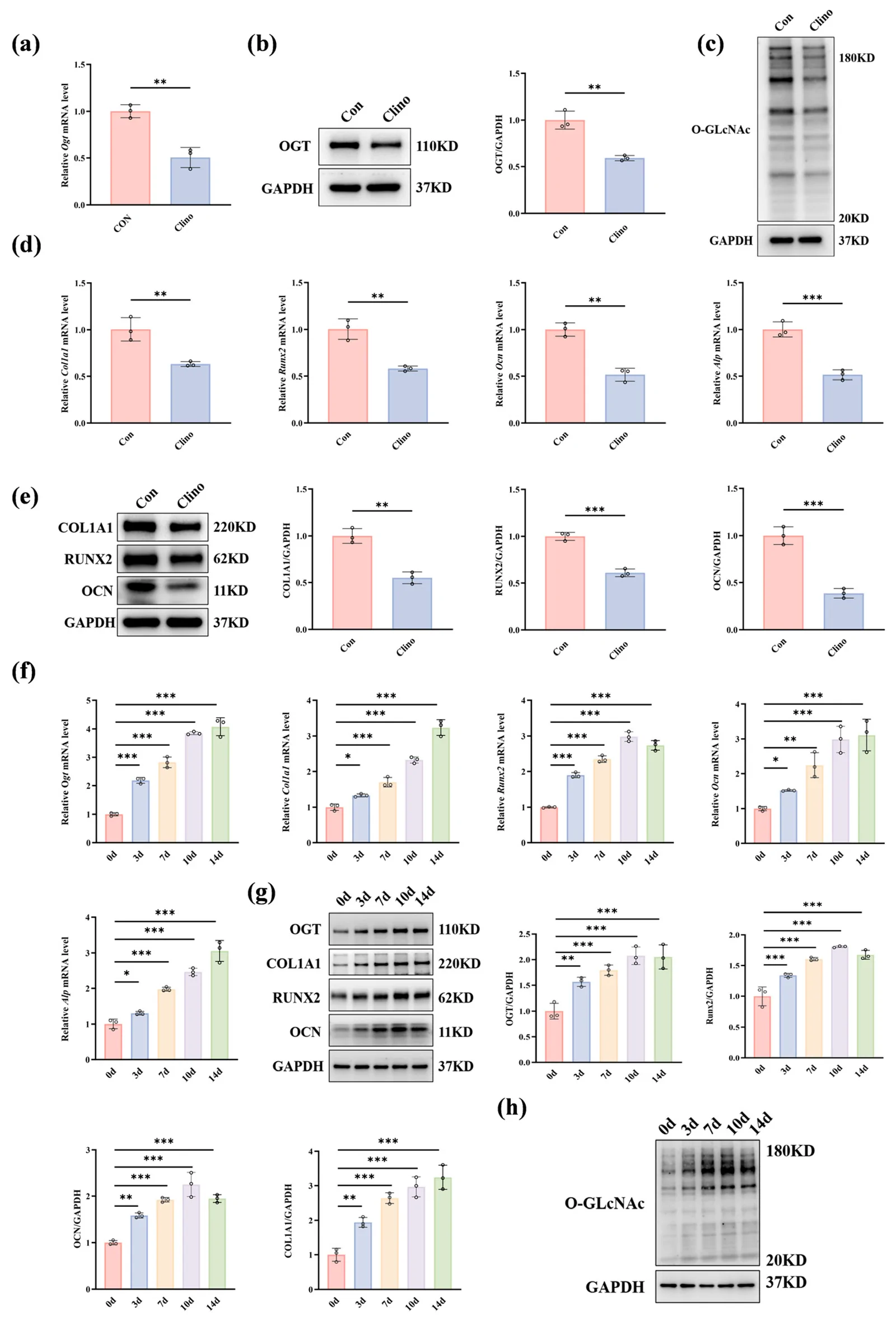
OGT and global O-GlcNAcylation levels are positively correlated with osteogenic differentiation. (**a**) mRNA expression levels of *Ogt* in MC3T3-E1 cells under unloading (n = 3). (**b**) Protein expression levels of OGT in MC3T3-E1 cells under unloading (n = 3). (**c**) Protein expression levels of O-GlcNAc in MC3T3-E1 cells under unloading (n = 3). (**d**) mRNA expression levels of *Col1a1, Runx2, Ocn, and Alp* in MC3T3-E1 cells under unloading (n = 3). (**e**) Protein expression levels of COL1A1, RUNX2 and OCN in MC3T3-E1 cells under unloading (n = 3). (**f**) mRNA expression levels of *Ogt, Col1a1, Runx2, Ocn, and Alp* in MC3T3-E1 cells during osteoblast differentiation by qRT-PCR (n = 3). (**g**) Protein expression levels of OGT, COL1A1, RUNX2 and OCN in MC3T3-E1 cells during osteoblast differentiation by western blotting (n = 3). (**h**) Protein expression levels of O-GlcNAc in MC3T3-E1 cells during osteoblast differentiation (n = 3). * *p* < 0.05, ** *p* < 0.01, *** *p* < 0.001.

Reduced mechanical stimuli on bones were the main cause of disuse osteoporosis. Given that O-GlcNAc modification played an important role in osteogenesis, we first examined the expression changes of OGT and overall O-GlcNAcylation under mechanical unloading using a two-dimensional clinostat. As shown in Figure 1a, OGT mRNA levels were significantly decreased after 48 h of unloading exposure compared with the static control group. Consistent with this, Western blotting analysis revealed a marked decrease in both OGT protein expression (Figure 1b) and global O-GlcNAcylation levels (Figure 1c). This downregulation was accompanied by downregulation of the osteogenic marker genes COL1A1, RUNX2, and OCN at both the mRNA and protein levels (Figure 1d,e). However, mRNA and protein expression of OGA showed no statistically significant difference between the Control (Con) and Clinorotation (Clino) groups (Figure S1a,b), indicating a selective regulation of OGT by mechanical unloading.

To investigate the functional involvement of OGT and O-GlcNAcylation in osteoblastic commitment, MC3T3-E1 cells were cultured in osteogenic induction medium to induce their differentiation into osteoblasts. Over the course of differentiation, the mRNA expression of osteogenic markers (*Col1a1*, *Runx2*, *Ocn* and *Alp*) was upregulated during differentiation alongside *Ogt* mRNA (Figure 1f). Western blotting analysis confirmed this trend at the protein level (Figure 1g). Moreover, a rise in global O-GlcNAcylation was also observed (Figure 1h). These observations suggest that OGT and O-GlcNAcylation may be involved in the osteogenic differentiation process.

### 3.2. OGT Promotes Osteogenic Differentiation and Mineralization

**Figure 2:**
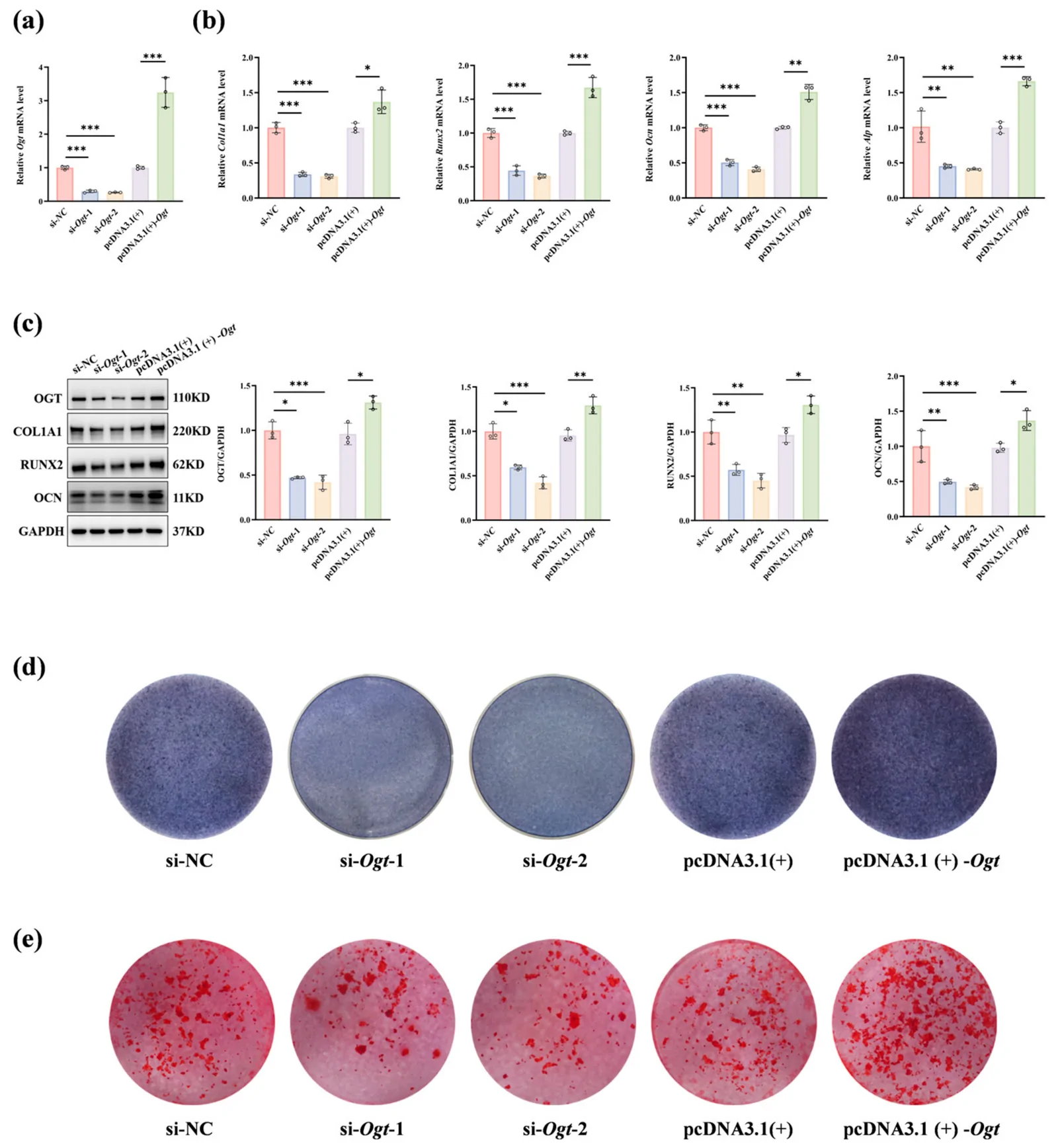
OGT promotes osteogenic differentiation and mineralization. (**a**) mRNA expression levels of *Ogt* after knockdown or overexpression of *Ogt* in MC3T3-E1 cells (n = 3). (**b**) mRNA expression levels of Col1a1, Runx2, Ocn, and Alp after knockdown or overexpression of *Ogt* in MC3T3-E1 cells (n = 3). (**c**) Protein expression levels of OGT, COL1A1, RUNX2 and OCN after knockdown or overexpression of *Ogt* in MC3T3-E1 cells (n = 3). (**d**) Representative images of ALP staining in MC3T3-E1 cells (n = 3). (**e**) Representative images of alizarin red S staining in MC3T3-E1 cells (n = 3). * *p* < 0.05, ** *p* < 0.01, *** *p* < 0.001.

To further investigate the regulatory role of OGT in vitro, MC3T3-E1 cells were transfected with si-*Ogt* or pcDNA3.1(+)-*Ogt*, and their differentiation and mineralization capabilities were assessed after osteogenic induction. Two efficient siRNAs targeting *Ogt* were identified to ensure experimental accuracy, and the knockdown or overexpression efficiency was verified by qRT-PCR and western blotting (Figure 2a,c). To rule out nonspecific cytotoxicity, cell viability was assessed by CCK-8 assay 48 h after transfection. OGT knockdown did not significantly impair viability relative to the negative control group (Supplementary Figure S2e), confirming that impaired osteogenic differentiation was not due to cellular toxicity. We then examined the expression of key osteogenic differentiation markers, including *Col1a1*, *Runx2*, *Ocn* and *Alp*. Knockdown of *Ogt* significantly reduced both mRNA and protein expression of these markers compared to the negative control group, whereas transfection with pcDNA3.1(+)-*Ogt* markedly increased their levels compared to the pcDNA3.1(+) group (Figure 2b,c). Furthermore, ALP staining and Alizarin Red S staining were performed to evaluate osteoblast differentiation and mineralization capacity. Compared with the negative control group, *Ogt* silencing significantly suppressed both differentiation and mineralization of MC3T3-E1 cells, while *Ogt* overexpression promoted these processes (Figure 2d,e). Collectively, these findings indicate that OGT is mechanoresponsive and OGT promotes osteoblast differentiation and mineralization.

### 3.3. OGT Partially Counteracts the Adverse Effects of Unloading on Osteoblast Differentiation

**Figure 3:**
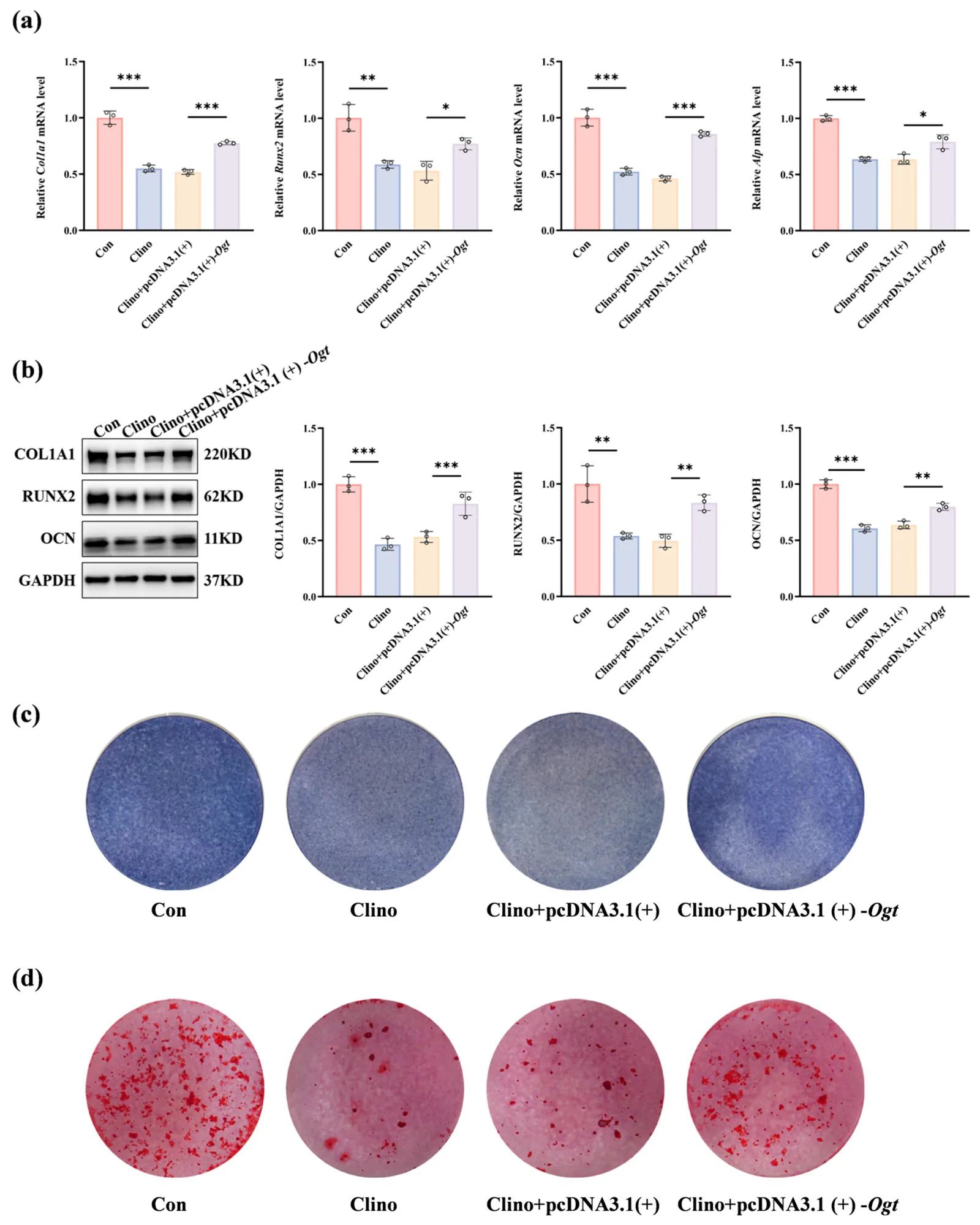
OGT partially counteracts the adverse effects of unloading on osteoblast differentiation. (**a**) mRNA expression levels of Col1a1, Runx2, Ocn, and Alp after overexpression of *Ogt* under mechanical unloading for 48 h (n = 3). (**b**) Protein expression levels of COL1A1, RUNX2 and OCN after overexpression of *Ogt* under mechanical unloading for 48 h (n = 3). (**c**) Representative images of ALP staining in MC3T3-E1 cells (n = 3). (**d**) Representative images of alizarin red S staining in MC3T3-E1 cells (n = 3). * *p* < 0.05, ** *p* < 0.01, *** *p* < 0.001.

To investigate whether OGT plays a role in osteoblast responses to mechanical unloading and whether overexpression of *Ogt* can alleviate the inhibition of osteogenic differentiation induced by unloading, MC3T3-E1 cells were transfected with pcDNA3.1(+)-*Ogt* or the corresponding empty vector and then exposed to 2D clinorotation for 48 h. qRT-PCR and western blotting analyses revealed that, compared with the control group, simulated unloading significantly reduced both the mRNA and protein levels of osteogenic markers COL1A1, RUNX2, and OCN, as well as the mRNA level of *Alp* (Figure 3a,b). Notably, *Ogt* overexpression partially rescued the impaired osteogenic differentiation induced by unloading (Figure 3a,b). Moreover, ALP and Alizarin Red S staining consistently showed that OGT overexpression markedly alleviated the suppression of osteoblast differentiation and mineralization caused by mechanical unloading (Figure 3c,d). Taken together, these findings suggest that OGT serves as a critical mediator of unloading-induced suppression of osteoblast differentiation.

### 3.4. OGT-Mediated O-GlcNAcylation Regulates Osteoblast Differentiation and Mineralization

**Figure 4:**
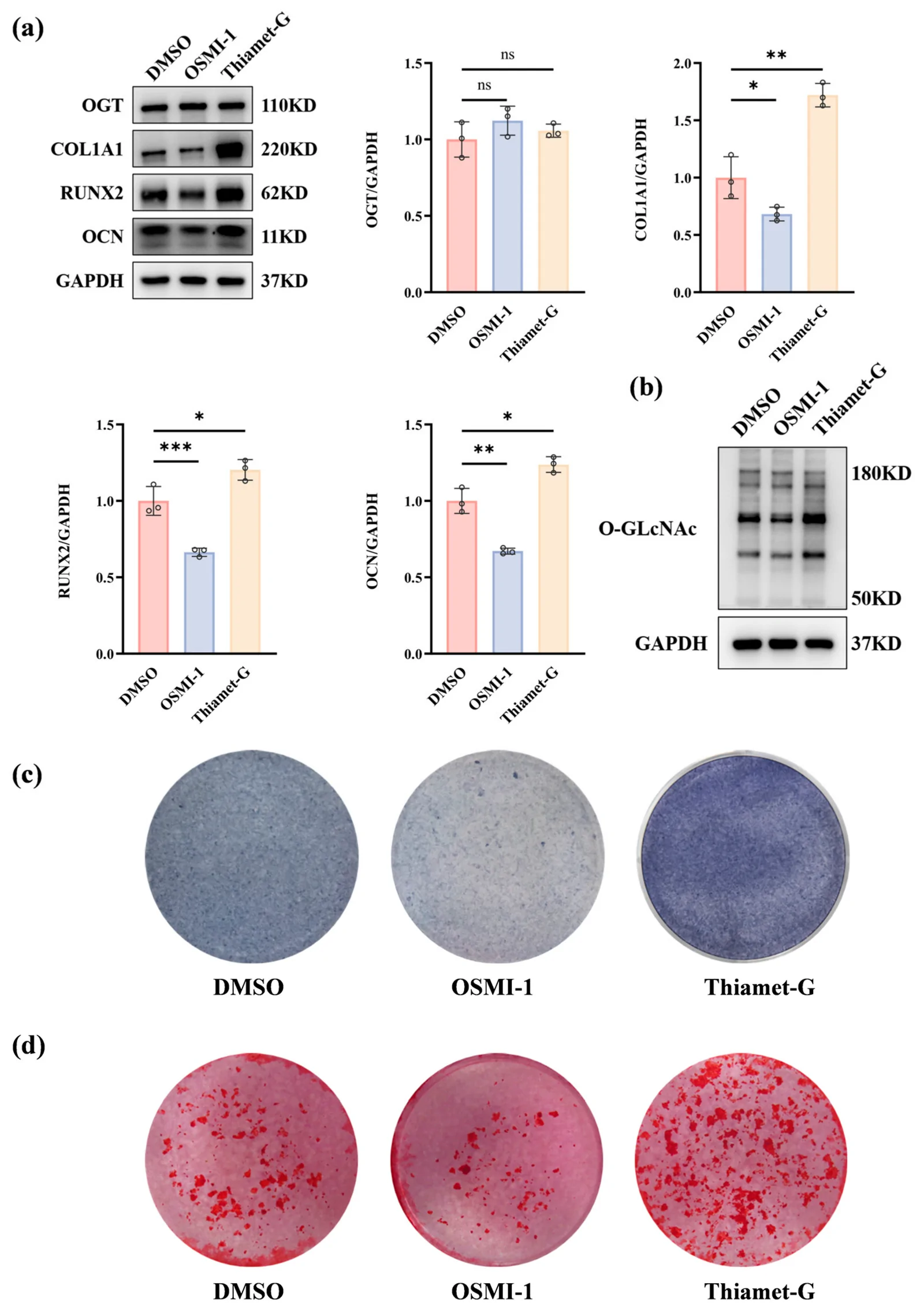
OGT-mediated O-GlcNAcylation regulates osteoblast differentiation and mineralization. (**a**) Protein expression levels of OGT, COL1A1, RUNX2 and OCN in MC3T3-E1 cells under OSMI-1 or Thiamet-G treatment (n = 3). (**b**) Protein expression levels of O-GlcNAc in MC3T3-E1 cells under OSMI-1 or Thiamet-G treatment (n = 3). (**c**) Representative images of ALP staining in MC3T3-E1 cells (n = 3). (**d**) Representative images of alizarin red S staining in MC3T3-E1 cells (n = 3). * *p* < 0.05, ** *p* < 0.01, *** *p* < 0.001. ns = no significance.

To determine whether the pro-osteogenic effect of OGT depends on its glycosyltransferase activity, MC3T3-E1 preosteoblasts were treated with pharmacological modulators of the O-GlcNAc cycle: OSMI-1 (a selective, cell-permeable OGT inhibitor that blocks O-GlcNAc addition) and Thiamet-G (a potent, selective OGA inhibitor that prevents O-GlcNAc removal). Prior to these experiments, dose–response and CCK-8 assays were performed to determine the optimal working concentrations and to exclude cytotoxic effects. At 10 μM OSMI-1 and 25 μM Thiamet-G, cell viability was not significantly affected after 24 h of treatment (Figure S2a–d). Treatment with OSMI-1 (10 μM) for 24 h markedly reduced global O-GlcNAcylation levels and suppressed the expression of osteogenic marker genes (Figure 4a,b), accompanied by markedly decreased ALP activity and impaired extracellular matrix mineralization (Figure 4c,d). In contrast, elevating O-GlcNAc levels with Thiamet-G (25 μM) treatment upregulated osteogenic gene expression, including COL1A1, RUNX2 and OCN (Figure 4a,b), and promoted osteogenic differentiation, as evidenced by increased ALP staining intensity and robust calcium deposition (Figure 4c,d). Notably, neither OSMI-1 nor Thiamet-G treatment affects OGT expression (Figure 4a). Together, these results demonstrate that OGT exerts its pro-osteogenic function strictly through its catalytic activity, and that dynamic modulation of O-GlcNAc homeostasis is sufficient to bidirectionally regulate osteoblast differentiation and mineralization.

To determine whether RUNX2 is a direct substrate of O-GlcNAcylation, we performed RUNX2 immunoprecipitation followed by immunoblotting with the O-GlcNAc-specific antibody RL2. Robust O-GlcNAcylation of RUNX2 was detected in MC3T3-E1 cells (Figure S3a). However, mechanical unloading did not significantly alter the RL2 signal intensity associated with immunoprecipitated RUNX2 relative to controls (Figure S3b). These findings indicate that RUNX2 O-GlcNAcylation is unlikely to serve as the principal mechanistic link through which the OGT/O-GlcNAc axis modulates osteogenic differentiation in response to mechanical unloading.

### 3.5. Thiamet-G Partially Rescues OGT Knockdown-Induced Impairment of Osteoblast Differentiation and Mineralization

To determine whether the O-GlcNAc modification level, rather than other non-catalytic or scaffolding functions of OGT, serves as the primary mechanistic driver of osteogenesis, we tested whether pharmacological inhibition of OGA with Thiamet-G could rescue the osteogenic defects caused by OGT knockdown. MC3T3-E1 preosteoblasts were transfected with either si-OGT or negative control siRNA (si-NC), and then treated with DMSO or Thiamet-G (25 μM) for 24 h.

**Figure 5:**
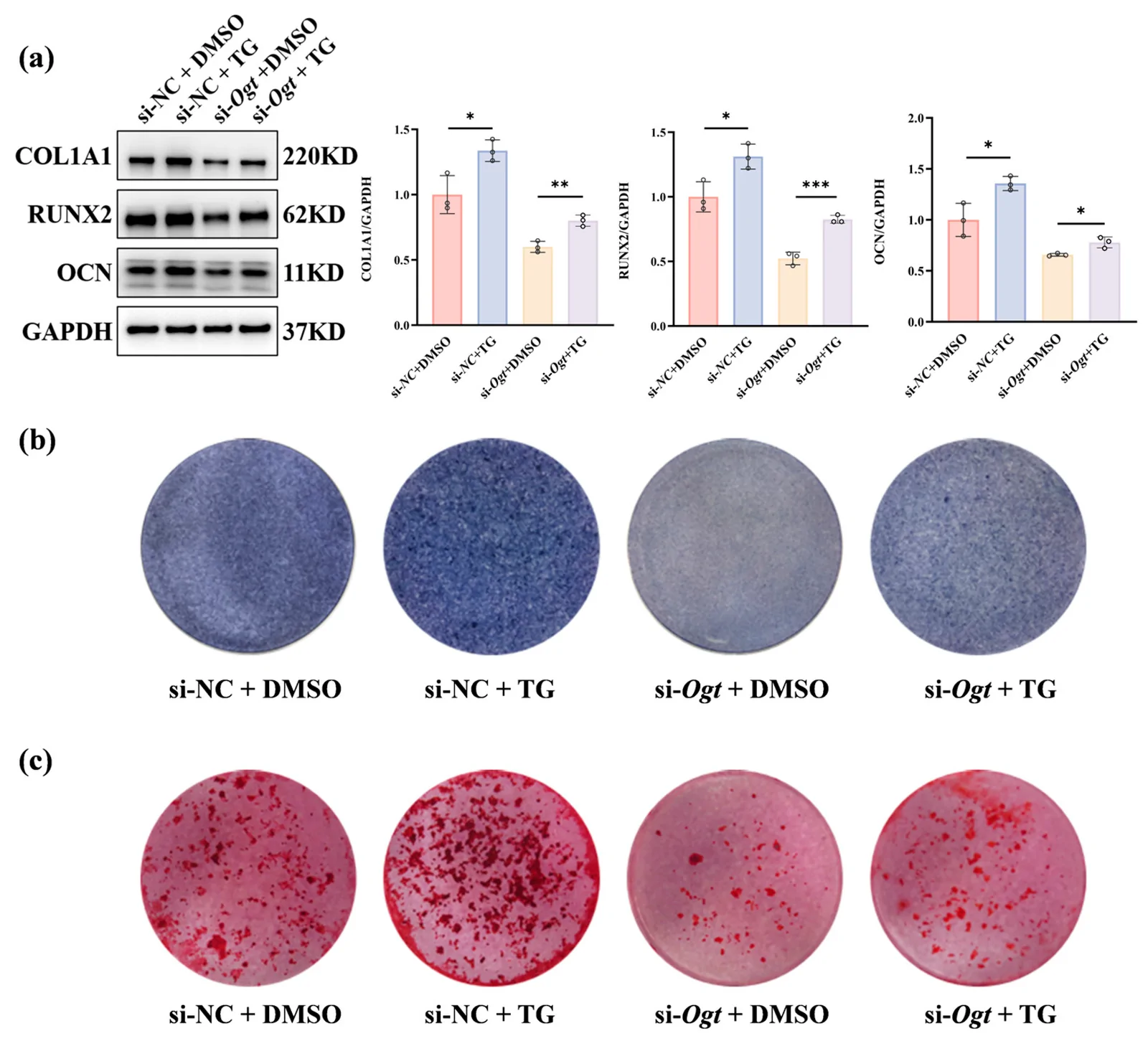
Thiamet-G partially rescues OGT knockdown-induced impairment of osteoblast differentiation and mineralization. (**a**) Protein expression levels of COL1A1, RUNX2 and OCN after Thiamet-G treatment in OGT knockdown MC3T3-E1 cells (n = 3). (**b**) Representative images of ALP staining in MC3T3-E1 cells (n = 3). (**c**) Representative images of alizarin red S staining in MC3T3-E1 cells (n = 3). * *p* < 0.05, ** *p* < 0.01, *** *p* < 0.001.

As expected, OGT knockdown decreased the expression of osteogenic differentiation markers, including COL1A1, RUNX2 and OCN, whereas Thiamet-G treatment reversed this trend (Figure 5a). Functionally, Thiamet-G significantly improved ALP staining intensity (Figure 5b) and calcium deposition (Figure 5c) in si-OGT-transfected cells. Notably, Thiamet-G treatment alone also promoted osteogenic differentiation and mineralization in control cells (Figure 5b,c). These findings indicate that elevating global O-GlcNAcylation levels through OGA inhibition can bypass OGT deficiency and partially restore osteogenic capacity, reinforcing O-GlcNAcylation as the critical downstream effector of OGT in this context.

## 4. Discussion

Bone loss induced by mechanical unloading poses a major clinical challenge, frequently observed in astronauts during prolonged space missions and in patients with restricted mobility. Accumulating evidence indicates that mechanical unloading disrupts skeletal homeostasis by impairing osteoblast-mediated bone formation while simultaneously enhancing osteoclastic resorption [2]. As the primary bone-forming cells, osteoblasts are intrinsically equipped to perceive mechanical stimuli and transduce them into intracellular biochemical signals that orchestrate transcriptional programs governing differentiation and matrix mineralization [39,40]. Nevertheless, the precise molecular machinery by which mechanical unloading compromises osteoblast function remains incompletely defined. In this study, we employed a 2D clinostat to simulate the mechanical unloading environment as an experimental model to investigate the mechanisms underlying disuse osteoporosis. Our data revealed that OGT expression and global O-GlcNAcylation were both downregulated in MC3T3-E1 osteoblasts subjected to clinorotation, whereas ectopic OGT overexpression partially ameliorated the suppressive effects of unloading on osteoblast differentiation and mineralization. Pharmacological blockade of OGT with OSMI-1 recapitulated the inhibitory phenotype observed under unloading conditions, whereas OGA inhibition with Thiamet-G exerted the opposite effect. These contrasting phenotypes establish a functional requirement for O-GlcNAcylation in regulating osteoblast differentiation under mechanical unloading. Furthermore, administration of Thiamet-G to OGT-deficient osteoblasts partially restored their differentiation and mineralization capacity. This demonstrates that elevated O-GlcNAc levels can compensate for diminished OGT catalytic activity, substantiating the involvement of O-GlcNAcylation in unloading-induced osteogenic impairment.

Post-translational modifications, including phosphorylation, ubiquitination, acetylation, lactylation, and glycosylation, dynamically regulate protein conformation, stability, subcellular localization and interactions, thereby participating in the control of bone metabolism [21]. While phosphorylation and ubiquitination have long dominated PTM research in bone biology [41,42,43,44,45], emerging studies highlight O-GlcNAcylation as a metabolically sensitive, nutrient-responsive regulator of skeletal development and homeostasis [46]. However, mechanistic insights into how O-GlcNAcylation influences bone pathophysiology, particularly in mechanically compromised contexts, remain relatively limited, and its role in disuse bone loss has yet to be explored. Zhang et al. [47] reported that O-GlcNAcylation activates RUNX2 to promote osteogenic differentiation of bone marrow stromal progenitors; similarly, O-GlcNAcylation of BSP promotes osteogenic differentiation of bone marrow mesenchymal stem cells [48]. Nevertheless, conflicting observations regarding the role of O-GlcNAcylation in osteogenic commitment have also emerged. For instance, under hyperglycemic conditions in diabetic patients, O-GlcNAcylation may become aberrantly elevated, and increased O-GlcNAc modification has been shown to diminish BMP2-induced osteogenic differentiation in C2C12 cells [35]. Notably, no prior work has systematically characterized OGT expression dynamics or global O-GlcNAcylation status in osteoblasts under mechanical unloading, nor has the functional relevance of this modification been experimentally validated in unloading conditions. Our data fill this gap by revealing consistent downregulation of OGT and O-GlcNAcylation in unloaded osteoblasts. These observations align with the established pro-osteogenic functions of physiological O-GlcNAcylation, as reported by Zhang et al. [47] and Wang et al. [48]. In contrast, the inhibitory effects observed under chronic hyperglycemia [35] highlight that dysregulated O-GlcNAc elevation, rather than O-GlcNAcylation per se, can impair osteoblast function. Collectively, these findings reflect the context-dependent and substrate-specific nature of O-GlcNAc signaling, in which tightly regulated O-GlcNAc homeostasis, not merely O-GlcNAc levels, is critical for sustaining osteogenic competence. Importantly, OGA levels remained unaltered under the same conditions, indicating that the reduction in O-GlcNAc modification is unlikely to result from enhanced deglycosylation but rather from diminished OGT-mediated glycosylation. Functionally, siRNA-mediated knockdown of OGT recapitulated the inhibitory effects of unloading on osteogenic differentiation and mineralization, whereas OGT overexpression promoted these processes, providing evidence that OGT functions as a positive regulator of osteoblast differentiation.

Recent advances position OGT and O-GlcNAcylation as central integrators of mechanotransduction across diverse tissues. OGT-mediated O-GlcNAcylation is critically involved in mechanical allodynia, as its inhibition alleviates neuropathic pain in mice [49], while mechanical compression induces chondrocyte hypertrophy by enhancing O-GlcNAcylation of Runx2 via a GLUT1-dependent mechanism [38]. Most compellingly, Chen et al. [50] demonstrated that Piezo1-mediated Ca^2+^ influx activates YAP signaling to enhance OGT activity in vascular smooth muscle cells, leading to Runx2 O-GlcNAcylation and pathological osteogenic trans-differentiation—a mechanism contributing to diabetic vascular calcification. Together, these findings suggest that OGT and O-GlcNAcylation may serve as a common mechanistic component involved in mechano-responsiveness, yet their involvement in disuse-related skeletal disorders has remained unexplored until now. Our study demonstrated that simulated mechanical unloading significantly suppresses both OGT mRNA and protein expression in MC3T3-E1 preosteoblasts, concomitant with reduced global O-GlcNAcylation and impaired osteogenic differentiation. Crucially, exogenous restoration of OGT expression partially rescues these deficits. O-GlcNAc depletion via the OGT inhibitor OSMI-1 recapitulated the inhibitory effects of unloading on osteoblast differentiation and mineralization. Conversely, pharmacological inhibition of O-GlcNAc turnover using the OGA inhibitor Thiamet-G elevated intracellular O-GlcNAcylation and promoted osteoblast differentiation and mineralization. Notably, neither OSMI-1 nor Thiamet-G altered total OGT protein abundance, indicating that phenotypic changes stem from perturbations in O-GlcNAc homeostasis rather than alterations in the enzyme’s protein level. Although the upstream mechanosensory pathways regulating OGT transcription, such as mechanosensitive miRNAs or the Hippo-YAP pathway, remain incompletely defined, our findings establish, for the first time, that the OGT/O-GlcNAc axis may participate in the mechanotransduction of disuse bone loss.

Thiamet-G is a selective, cell-permeable inhibitor of O-GlcNAcase (OGA) that enhances global O-GlcNAcylation by blocking enzymatic deglycosylation. Prior studies have shown that Thiamet-G attenuates RANKL- and M-CSF-driven osteoclastogenesis in RAW264.7 macrophages via O-GlcNAc elevation [51]. In a human TNFα-driven murine model of rheumatoid arthritis, Thiamet-G improved trabecular microarchitecture, reduced focal bone resorption, and mitigated both local and systemic bone loss [52]. Emerging evidence further supports its pro-osteogenic role: in pulp repair models, Thiamet-G promoted reparative dentinogenesis, evidenced by upregulated BMP2, Runx2, and Ocn expression and enhanced alkaline phosphatase (ALP) activity [53]. Notably, in a diabetic vascular calcification model, Thiamet-G treatment restored O-GlcNAcylation and osteogenic marker (Runx2 and BMP2) expression despite Piezo1 inhibition, indicating that pharmacological elevation of O-GlcNAc levels can functionally compensate for upstream Piezo1/OGT suppression [50]. However, the role of Thiamet-G in disuse osteoporosis has not been previously reported. In the present study, we demonstrate that Thiamet-G effectively rescues osteogenic marker expression, as well as osteoblast differentiation and mineralization capacity in the context of OGT knockdown. This observation expands the potential application of Thiamet-G to disuse bone loss and uncovers a distinctive mode of action whereby it bypasses OGT deficiency by sustaining cellular O-GlcNAc levels.

Although Thiamet-G treatment confirmed that elevating global O-GlcNAc levels is sufficient to rescue osteogenic differentiation, the critical downstream effectors mediating this effect remain undefined. We therefore focused on RUNX2—a master transcriptional regulator of osteogenesis and a well-established O-GlcNAc substrate. Under basal conditions, RUNX2 exhibited constitutive O-GlcNAcylation (Figure S3a); however, mechanical unloading did not significantly alter its RL2 immunoreactivity relative to static controls (Figure S3b). This observation does not preclude a functionally relevant role for RUNX2, as the RL2 antibody detects total O-GlcNAc modification and lacks site-specific resolution. Notably, RUNX2 harbors multiple experimentally verified O-GlcNAcylation sites that are spatially adjacent to key phospho-regulatory residues. At these loci, O-GlcNAcylation and phosphorylation may dynamically compete or synergize to fine-tune transcriptional activity. Thus, mechanical unloading could selectively modulate modification at functionally critical sites without affecting overall O-GlcNAc level. Even if RUNX2 is not the primary direct effector, O-GlcNAcylation may regulate osteogenesis through alternative mechanisms, including modulation of other transcription factors, epigenetic regulators, or extracellular vesicle (EV)-mediated intercellular communication [33,35,37,54]. Crucially, our central conclusion does not depend on RUNX2 or any single molecular target. Instead, it rests on functional evidence: genetic and pharmacological manipulation of OGT activity demonstrated that global O-GlcNAc levels are both necessary and sufficient to sustain osteogenic differentiation. Therefore, the unaltered bulk O-GlcNAcylation of RUNX2 under unloading does not undermine the mechanistic validity of our core finding. Future work employing chemoenzymatic labeling coupled with mass spectrometry or site-specific O-GlcNAc antibodies will be essential to map stimulus-dependent changes in RUNX2 modification stoichiometry and occupancy.

Several limitations of this study should be acknowledged. First, all experiments were performed in the MC3T3-E1 pre-osteoblast cell line using an in vitro clinostat system; validation in primary BMSCs and in vivo tail-suspension models is needed. Notably, bone homeostasis is governed by intricate multicellular crosstalk among osteoblasts, osteocytes, and osteoclasts. While our study specifically addresses intrinsic osteoblast-lineage mechanisms, it does not extend to the regulation of the OGT/O-GlcNAc axis in osteocytes or its functional impact on osteoclastogenesis. To bridge this gap, future work will prioritize validation in the MLO-Y4 osteocyte model and assessment of osteoclastogenic effects via RANKL/OPG signaling or co-culture systems. Second, the absence of O-GlcNAcome profiling by mass spectrometry precludes a comprehensive definition of the downstream effector protein network regulated by this axis. Nevertheless, these limitations do not undermine the core conclusions of our work: mechanical unloading disrupts intracellular O-GlcNAc homeostasis primarily through OGT downregulation, and this disruption is a functionally significant driver of impaired osteogenic differentiation. Pharmacological preservation of O-GlcNAc levels exerted a clear rescue effect. This study systematically delineates the OGT/O-GlcNAc axis as a pivotal regulator of osteoblast dysfunction under mechanical unloading. These findings hold translational relevance for clinical populations experiencing skeletal disuse, including elderly patients undergoing prolonged bed rest, individuals with paralysis following spinal cord injury, and those immobilized post-fracture fixation. In such settings, diminished mechanical loading likely contributes to rapid bone loss, at least in part via suppression of the OGT/O-GlcNAc pathway. Thus, maintaining or pharmacologically restoring O-GlcNAc homeostasis holds promise as a chemical adjunctive intervention strategy, complementing physical rehabilitation approaches such as vibration training or functional electrical stimulation, particularly for patients with disuse bone loss who are unable to engage in active or passive exercise.

## 5. Conclusions

In conclusion, this study identifies a novel mechano-metabolic axis involved in disuse osteoporosis. This axis involves mechanical unloading-induced downregulation of OGT and reduced global O-GlcNAcylation, which impairs osteoblast differentiation and mineralization. Conversely, restoring O-GlcNAc homeostasis through OGT overexpression or OGA inhibition rescues these defects, even under unloading conditions. Collectively, our findings establish the OGT/ O-GlcNAc axis as a key nexus linking mechanical stimuli to osteogenic commitment and point to this pathway as a promising therapeutic strategy for combating disuse osteoporosis. Future studies should validate this axis in vivo, elucidate the upstream mechanosensing mechanisms and downstream effector networks, and explore pharmacological strategies to modulate O-GlcNAc homeostasis for clinical translation.

## Figures and Tables

**Table 1:** The sequence of primers.

Name	Sequence (5′~3′)
*Ogt*-F	GGCTATGTGAGTTCTGACTTCGG
*Ogt*-R	GATTGGCTTCCGCCATCACCTT
*Oga*-F	ACTGTGCCAACAGGACCATCCT
*Oga*-R	CTGTGCTGAAGAGTGACTACGAC
*Col1a1*-F	AGGCGAACAAGGTGACAGAGG
*Col1a1*-R	GGAGAACCAGGAGAACCAGGAG
*Runx2*-F	CGGCAAGATGAGCGACGTGAG
*Runx2*-R	TGCTGCTGCTGCTGCTGTTG
*Osx*-F	TCGTCTGACTGCCTGCCTAGTG
*Osx*-R	CTGCGTGGATGCCTGCCTTG
*Ocn*-F	CCTCAGTCCCCAGCCCAGATC
*Ocn*-R	TGCGTTTGTAGGCGGTCTTCAAG
*Alp*-F	AACTGATGTGGAATACGAACTGCATG
*Alp*-R	CATAGTGGGAATGCTTGTGTCTGG
*Gapdh*-F	GACTCCACTCACGGCAAATTCAAC
*Gapdh*-R	GACACCAGTAGACTCCACGACATAC

**Table 2:** RNA Oligo sequences.

Name	Sequence (5′~3′)
si—NC sense	UUCUCCGAACGUGUCACGUTT
si—NC antisense	ACGUGACACGUUCGGAGAATT
si—*Ogt*-1 sense	GCACAGCUCUGAAACUUAATT
si—*Ogt*-1 antisense	UUAAGUUUCAGAGCUGUGCTT
si—*Ogt*-2 sense	GGCAGAUUCAGAUAACAAUTT
si—*Ogt*-2 antisense	AUUGUUAUCUGAAUCUGCCTT

## Data Availability

The original contributions presented in this study are included in the article/Supplementary Material. Further inquiries can be directed to the corresponding authors.

## Supplementary Materials

The following supporting information can be downloaded at https://www.mdpi.com/article/10.3390/cimb48090961/s1.

## Abbreviations

The following abbreviations are used in this manuscript:

ALP	Alkaline Phosphatase
BMP2	Bone Morphogenetic Protein 2
BMSCs	Bone Marrow Mesenchymal Stem Cells
BSP	Bone sialoprotein
Clino	Clinorotation
Col1a1	Collagen Type I Alpha 1 Chain
Con	Control
NC	Negative Control
OCN	Osteocalcin
OGA	O-GlcNAcase
O-GlcNAc	O-linked N-acetylglucosamine
O-GlcNAcylation	O-linked N-acetylglucosaminylation
OGT	O-GlcNAc transferase
qRT-PCR	Quantitative Real-time Polymerase Chain Reaction
RUNX2	Runt-Related Transcription Factor 2
TG	Thiamet-G
