# Supplementary material for: Mechanical Unloading Inhibits Osteoblast Differentiation via Downregulation of OGT-Mediated O-GlcNAcylation
Source: Curr Issues Mol Biol. 2026 Sep 20;48(9):961. doi: 10.3390/cimb48090961 (PMC13605618; doi:10.3390/cimb48090961)
Supplement: Supplementary file 1 [file cimb-48-00961-s001.zip › cimb-4499295-supplementary.pdf]

Supplementary Materials for

Mechanical Unloading Inhibits Osteoblast Differentiation via Downregulation of OGT-Mediated O-GlcNAcylation

Xiran Zhao [1,†], Junfei Zhang [1,†], Zhikui Li [1], Quan Sun [1], Liqun Xu [2], Tong Xue [1], Jiangdong Zhao [1], Xian Guo [1], Ru Zhang [1], Xuan Xie [1], Zhijun Yan [1], Zebing Hu[1], Shu Zhang[1,*], and Fei Shi[1,*]

*To whom correspondence should be addressed:

shuzhang@fmmu.edu.cn (S.Z); shifei719@fmmu.edu.cn (F.S).

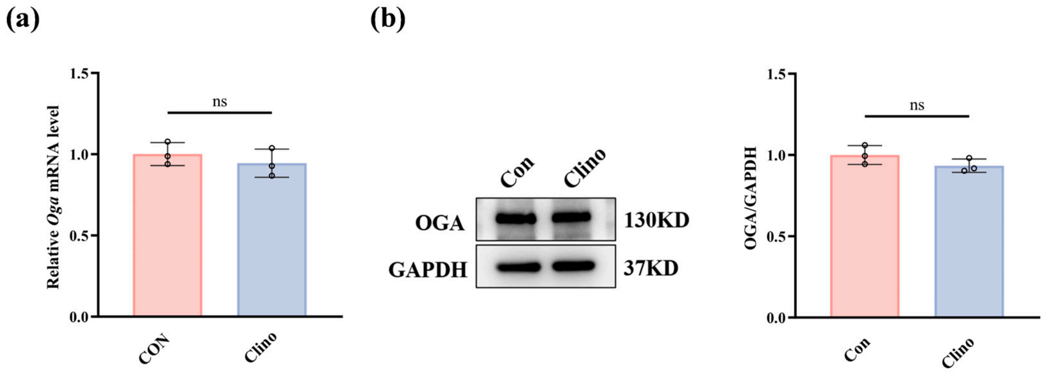

Figure S1. mRNA and protein expression of OGA under unloading. (a) mRNA expression levels of *Oga* in MC3T3-E1 cells under unloading (n = 3). (b) Protein expression levels of OGA in MC3T3-E1 cells under unloading (n = 3). ns = no significance.

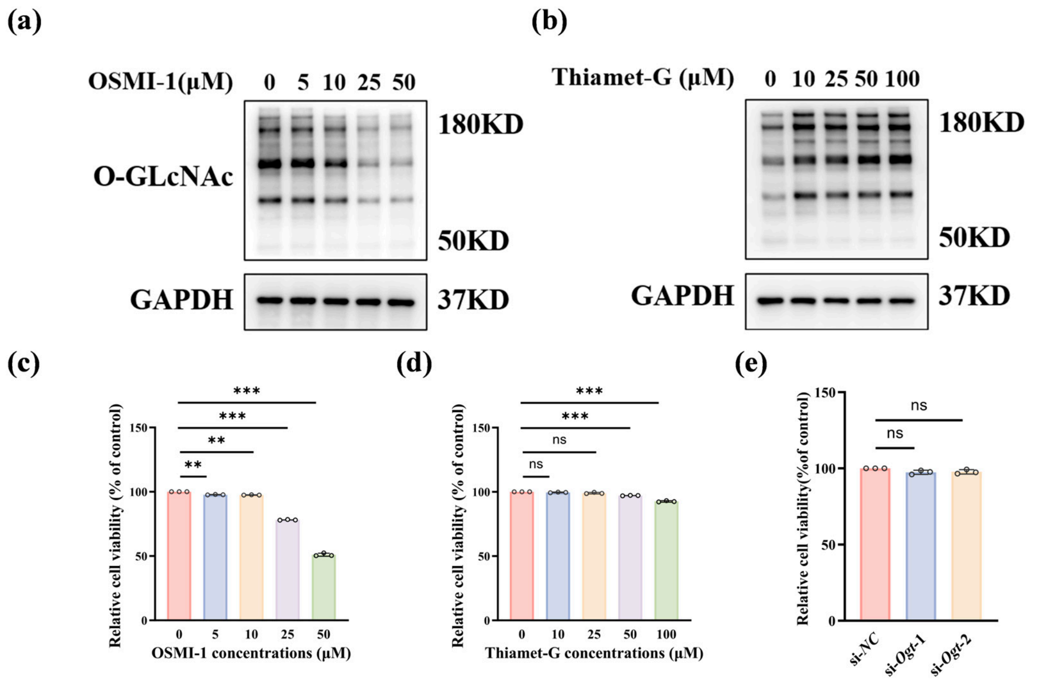

Figure S2. Effect of OSMI-1, Thiamet-G treatment and si-*Ogt* transfection in MC3T3-E1 cells. (a) Protein expression levels of global O-GlcNAc after OSMI-1 treatment in MC3T3-E1 cells (n = 3). (b) Protein expression levels of global O-GlcNAc after Thiamet-G treatment in MC3T3-E1 cells (n = 3). (c) MC3T3-E1 cell viability after 24 h OSMI-1 treatment (n = 3). (d) MC3T3-E1 cell viability after 24 h Thiamet-G treatment (n = 3). (e) MC3T3-E1 cell viability after 48 h si-*Ogt* transfection (n = 3). ns = no significance., $^{**}p < 0.01$, $^{***}p < 0.001$.

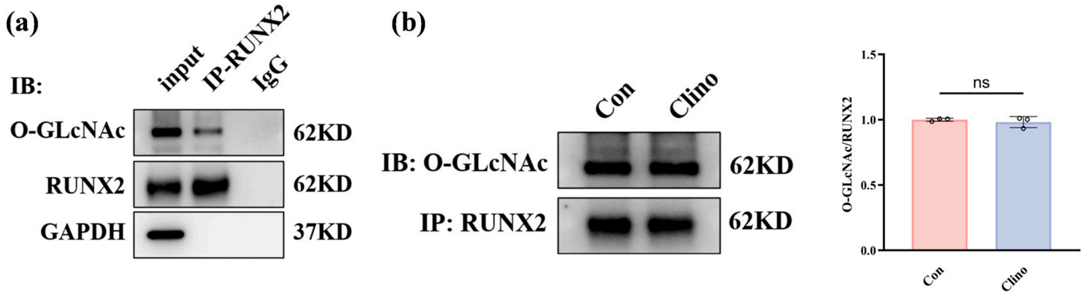

Figure S3. RUNX2 immunoprecipitation and O-GlcNAcylation quantification in MC3T3-E1 cells. (a) Protein expression levels of O-GlcNAc after RUNX2 immunoprecipitation in MC3T3-E1 cells. (n = 3). (b) Protein expression levels of Runx2 O-GlcNAcylation in MC3T3-E1 cells under unloading (n = 3). ns = no significance.
